# Sequence analysis of genes mediating extended-spectrum beta-lactamase (ESBL) production in isolates of *Enterobacteriaceae* in a Lagos Teaching Hospital, Nigeria

**DOI:** 10.1186/s12879-015-1005-x

**Published:** 2015-07-07

**Authors:** Muhabat Adeola Raji, Wafaa Jamal, Omoh Ojemeh, Vincent Olubunmi Rotimi

**Affiliations:** Department of Medical Microbiology and Parasitology, Lagos State University College of Medicine/Lagos State University Teaching Hospital, Ikeja, Nigeria; Department of Microbiology, Faculty of Medicine, Kuwait University, Kuwait City, Kuwait; Microbiology Laboratory, BT Health and Diagnostic Centre, Lagos State University Teaching Hospital, Ikeja, Nigeria

**Keywords:** Multidrug resistance, ESBL, *Enterobacteriaceae*, Southwest Nigeria, CTX-M-15

## Abstract

**Background:**

Extended-spectrum β-lactamases (ESBLs) in Gram-negative organisms is now a major concern in *Enterobacteriaceae* worldwide. This study determined a point-prevalence and genetic profiles of ESBL-producing isolates among members of the family *Enterobacteriaceae* in Lagos State University Teaching Hospital Ikeja, Nigeria.

**Methods:**

Consecutive non-repetitive invasive multidrug-resistant isolates of the family *Enterobacteriaceae* obtained over a period of 1 month (October 2011) were studied. The isolates were identified using VITEK-2/VITEK MS Systems. Susceptibility testing was performed using E test technique; results were interpreted according to the criteria recommended by the Clinical and Laboratory Standards Institute (CLSI, 2012). ESBL production was detected by E test ESBL method and confirmed by polymerase chain reaction (PCR).

**Results:**

During the one-month study period, 38 isolates with ESBL phenotypic characteristics were identified and confirmed by PCR. Of these, 21 (55.3 %) were *E. coli*, 12 (31.6 %) *K. pneumoniae*, 3 (7.9 %) *Proteus* spp., 1 (2.6 %) each *M. morganii* and *C. freundii*. Thirty (79 %) harbored *bla*_CTX-M_ genes. Sequence analysis revealed that they were all *bla*_CTX-M-15_ genes. Twenty-nine (96.7 %) of these, also harbored *bla*_TEM_ genes simultaneously. All the CTX-M-15-producing isolates carried insertion sequence *bla*_IS*EcP*1_ upstream of *bla*_CTX-M-15_ genes. The *E. coli* isolates were genetically heterogeneous, while the *K. pneumoniae* had 98 % homology.

**Conclusions:**

Our point-prevalence surveillance study revealed a high prevalence of *Enterobacteriaceae* isolates harboring *bla*_CTX-M-15_ in the Hospital. Urgent implementation of antibiotic stewardship and other preventive strategies are necessary at this time in our hospital.

## Background

Gram-negative organisms belonging to the family *Enterobacteriacae* commonly produce beta-lactamases, which confer resistance to most penicillins but not to expanded-spectrum cephalosporins. The genes encoding these beta-lactamases are plasmid-borne, and belong to the TEM-1, TEM-2, and SHV-1 types [1]. Infections caused by members of the family *Enterobacteriaceae* are treated with cephalosporins, particularly the third-and fourth-generation cephalosporins. However, resistance to these drugs has emerged throughout the world with widespread of resistant strains. By the middle of the 80s, resistance to the expanded-spectrum cephalosporin became evident and various studies have shown that the resistance was mediated by structural mutation in the older enzymes [1, 2].

In *Enterobacteriaceae*, resistance to cephalosporin is commonly due to production of extended-spectrum β-lactamases (ESBLs). The emergence of ESBLs in Gram-negative organisms is now a major concern worldwide [1], and the presence of these enzymes is among the most important resistance determinants to have emerged in *Enterobacteriaceae* [3–7]. Most ESBLs are derivatives of TEM and SHV β-lactamase families. Other groups such as PER and CTX-M types have been described [8, 9]. In addition, other β-lactamases, including those belonging to Ambler class B (metallo-β-lactamase), class A (e.g. KPC) or class D (OXA-48), capable of hydrolyzing carbapenems have emerged [10–12].

The literature is awash with evidence of global dissemination of CTX-M type ESBL of pandemic proportion [9, 13, 14]. The production of this enzyme is mediated by the *bla*_CTX-M_ gene, which confers resistance to the third-generation cephalosporins particularly in *Escherichia coli* and *Klebsiella* spp. [15]. Several phenotypic and genotypic studies have documented the emergence of CTX-M-type extended-spectrum β-lactamases as well as the genes encoding their production in *Enterobacteriaceae*, in Nigeria. However, most of these studies have been on randomly selected *E. coli* and *K. pneumoniae* [16–19] and *Salmonella enterica* serovar Typhi [20] and thus the true prevalence of CTX-M in most parts of the country is unknown. In our hospital, cephalosporins are first line antibiotics used in the treatment of Gram-negative sepsis and other infective conditions. A previous study conducted earlier in the same hospital demonstrated high resistance rates among clinically significant species of the family *Enterobacteriaceae* against the cephalosporins and other β-lactam antibiotics [21]. With such high resistance rates, it is conceivable that CTX-M would also be the dominant ESBL type responsible for this high level of resistance in our hospital.

This study was undertaken to investigate a point-prevalence and genetic profiles of ESBL-producing isolates among members of the family *Enterobacteriaceae* causing infections in patients on admission in a tertiary hospital in Lagos.

## Methods

### Bacterial isolates and setting

Thirty-eight consecutive isolates of multidrug-resistant invasive species of the family *Enterobacteriaceae* were obtained over a period of one month (October 2011), during routine laboratory investigation, from in-patients at the Lagos State University Teaching Hospital (LASUTH) located in Ikeja, a suburban part of Lagos. The hospital serves as a referral center for about 6 million Lagosians. It has one adult Intensive Care Unit (ICU), 1 Critical Care Unit (CCU), a dialysis unit and an oncology unit. The age, sex, nationality, previous hospital admissions, and documented travel history were all carefully noted. Duplicate isolates were omitted from the study.

The bacterial isolates were identified by VITEK-2 system (bioMérieux, Hazelwood, MO, USA). In addition, when necessary, further confirmation was carried out with VITEK MS, a matrix-assisted laser desorption ionization-time of flight (MALDI-TOF) mass spectrometry (MS) system (bioMerieux, Marcy-l’Etoile, France).

### Susceptibility testing

Susceptibility testing of the isolates was performed by determining the minimum inhibitory concentrations (MICs) of amikacin, amoxicillin-clavulanic acid, cefepime, cefotaxime, cefoxitin, ceftazidime, ciprofloxacin, colistin, ertapenem, imipenem, gentamicin, meropenem, piperacillin-tazobactam, and tigecycline on Mueller-Hinton agar plates using E test (bioMerieux) technique. A quality control strain, *Escherichia coli* ATCC 25922 was included in each run. The results were interpreted according to the break-points and criteria recommended by the Clinical and Laboratory Standards Institute (CLSI, 2012) [22].

### Confirmation of ESBL

All the ESBL-producing isolates were phenotypically detected by E test ESBL method using cefotaxime (CT)/cefotaxime combined with clavulanic acid (CTL) and ceftazidime (TZ)/ceftazidime combined with clavulanic acid (TZL) (bioMerieux) and were confirmed by polymerase chain reaction (PCR). In-house ESBL-producing *E. coli* strain K31 [23] and ESBL-negative strain were included in the test runs as positive and negative controls, respectively.

### PCR amplification and sequencing

PCR assays were carried out with a series of primers to detect the following genes mediating *bla*_SHV_, *bla*_TEM_, *bla*_CMY-6_, *bla*_CMY-4_, and *bla*_CTX-M_ [24]. PCR products were sequenced with a 3130xl Genetic Analyzer (Applied Biosystems, Hitachi High-Technologies Corporation, Tokyo, Japan). Sequences were compared and aligned with reference sequences available in the GenBank.

### Detection of insertion sequence, IS*Ecp1*

The genetic organization of the *bla*_IS*Ecp*1_ was investigated by sequencing this short segment using the following primers: IS*Ecp1A* (5’-GCA GGT CTT TTT CTG CTC C-3’) and IS*Ecp1B* (5’- ATT TCC GCA GCA GCA CCG TTT GC- 3’) [25].

### Genotyping of isolates

Fifteen randomly selected strains of the *bla*_CTX-M-15_-positive isolates (9 *E. coli* and 6 *K. pneumoniae*) were investigated for genetic relatedness using pulsed-field gel electrophoresis (PFGE) with *X*ba1 digestion of the genomic DNA separated by electrophoresis in 1.2 % agarose gel [26] and the strains compared by differences in number and mobility of the bands.

## Results

During this one month study, a total of 73 isolates belonging to the family *Enterobacteriaceae* were studied. Of these, 38 (52.1 %) were ESBL-producing isolates, 21 (55.3 %) of which were *E. coli*, 12 (31.6 %) *K. pneumoniae*, 3 (7.9 %) *Proteus* spp., 1 each (2.6 %) *M. morganii* and *Citrobacter freundii*. They were isolated from urine (24), wound swabs (8), blood culture (3) and respiratory secretions (3). These specimens were obtained from infected in-patients who were ethnic Nigerians, predominantly Yorubas. Their ages ranged from 6 – 89 years (mean 59.2 years); 20 (52.6 %) were males and 18 (47.4 %) females with a male-to-female ratio of 1.1:1.

### Prevalence of *bla*_CTX-M_ ESBL-positive *Enterobacteriaceae*

The ESBL-producing *E. coli* and *K. pneumoniae* were multidrug-resistant isolates (MDR) showing resistance to five or more antibiotics (Table 1). The number of MDR *P. mirabilis*, *M. morganii* and *C. freundii* isolates was too small for any analysis.

**Table 1:** Antibiotic susceptibility profiles of the *bla*_CTX-M_ ESBL-positive *E. coli and K. pneumoniae.*

Antibiotics	MIC (μg/ml) for the *bla*_CTX-M_-positive isolates:			
	*E. coli*		*K. pneumoniae*	
	(n = 18)		(n = 10)	
	MIC_50_	MIC_90_	MIC_50_	MIC_90_
Amikacin	2	16	0.5	4
Ampicillin	>256	>256	>256	>256
Amoxicillin-clavulanate	6	16	8	16
Aztreonam	>256	>256	>256	>256
Cefepime	>256	>256	>256	>256
Cefotaxime	>256	>256	>256	>256
Cefoxitin	2	12	4	12
Ceftazidime	32	>256	>256	>256
Cefuroxime	>256	>256	>256	>256
Ciprofloxacin	>32	>32	>32	>32
Colistin	0.002	2	0.064	1.5
Ertapenem	0.0125	0.75	0.064	0.5
Gentamicin	1	16	0.5	>256
Imipenem	0.125	0.75	0.125	0.25
Meropenem	0.094	0.125	0.047	1
Piperacillin-tazobactam	0.75	>256	0.5	>256
Tigecycline	0.5	1.5	0.125	1

As shown in Table 2, of the 38 ESBL-producing isolates, 30 (79 %) harbored *bla*_CTX-M_ genes. Sequence analysis of these genes revealed that they were all *bla*_CTX-M-15_. Twenty-nine (96.7 %) of these, also harbored *bla*_TEM_ genes simultaneously. A combination of *bla*_CTX-M-15_, *bla*_TEM_ and *bla*_SHV_ were found in 6 isolates; the distribution of the *bla*_SHV_ was *bla*_SHV-11_ (2), *bla*_SHV-12_ (2) and *bla*_SHV-112_ (2). All 30 isolates positive for *bla*_CTX-M_ carried insertion sequence *bla*_IS*EcP*1_ upstream of the *bla*_CTX-M-15_ genes.

**Table 2:** Distribution of *bla*_CTX-M_ β-lactamase genes among ESBL-producing Enterobacteriaceae isolates

Bacterial isolate	No. (%) of ESBL-positives (n = 38)	No. (%) of isolates harboring *bla* genes:		
		*bla*_CTX-M-15_	*bla*_TEM-1_	*bla*_SHV_
		(n = 30)	(n = 29)	(n = 6)
*E. coli*	21 (55.3)	18 (60.0)	17 (58.6)	2 (33.3)
*K. pneumoniae*	12 (31.6)	10 (33.3)	9 (31.0)	2 (33.3)
*P. mirabilis*	3 (7.9)	2 (6.7)	3 (10.4)	0 (0)
*M. morganii*	1 (2.6)	0 (0)	0 (0)	1 (16.7)
*C. freundii*	1 (2.6)	0 (0)	0 (0)	1 (16.7)

The ESBL E-test for ESBL production detected all the *bla*_CTX-M_, *bla*_TEM_ and *bla*_SHV_-positive isolates confirmed by PCR. Testing both cefotaxime and ceftazidime was necessary for detection of CTX-M-positive isolates. As demonstrated in Table 1, the MIC_90_s of the third-generation cephalosporins were all >256 μg/ml. Our *bla*_CTX-M_-positive isolates, with or without SHV and TEM, were susceptible to amikacin, colistin, imipenem, meropenem and tigecycline. Three isolates (2 *E. coli* and 1 *K. pneumoniae*) were not inhibited at the cut-off breakpoint (0.5 μg/ml) of ertapenem but were susceptible to imipenem (MIC = 0.125 and 0.5 μg/ml) and meropenem (0.5 and 0.75 μg/ml). There was no specific distribution of the CTX-M-15-positive isolates among ethnic groups of the Yoruba race. Two *E. coli* and a *Proteus* spp. isolates were positive for ESBL but the genes encoding their production were not detected.

### Clonal relatedness of the *bla*_CTX-M-15_ harboring isolates

The fingerprinting of the genomic DNA of the *bla*_CTX-M-15_-positive randomly selected isolates of *E. coli* and *K. pneumoniae* showed that the *E. coli* isolates were genetically heterogeneous, as the isolates did not fall within a particular cluster. On the other hand, there was about 98 % similarity with the *K. pneumoniae* isolates.

## Discussion

The predominant genotype of the ESBL found in the clinical isolates of *Enterobacteriaceae* in this study was *bla*_CTX-M_**-**type genes with all being *bla*_CTX-M-15_; over 96 % of these 38 isolates also harbored a *bla*_TEM_ β-lactamase gene. Five isolates co-harbored narrow-spectrum *bla*_SHV-11_ and other *bla*_SHV_ genes. Our data demonstrate a very high prevalence of *bla*_CTX-M_ type ESBL genes among the multidrug-resistant (MDR) isolates studied. This lends credence to the worldwide pandemic spread of the CTX-M β-lactamase enzyme, a phenomenon that has reached epidemic proportion among members of the family *Enterobacteriaceae*. The ESBL mediated by *bla*_CTX-M_ type β-lactamase genes are undoubtedly the most widespread enzymes produced among members of this family. This assertion is predicated on the fact that over 79 % of our ESBL-producing isolates harbored *bla*_CTX-M_, the gene that mediates CTX-M enzyme production. Remarkably, all the CTX-M enzymes were CTX-M-15, making this type of β-lactamase the most common type detected in *Enterobacteriaceae* in this Lagos hospital. The dominance of *bla*_CTX-M-15_ β-lactamase genes in our snap surveillance study confirms what other workers had earlier reported in North America [15, 27, 28], Europe [29, 30], South America [31] the Middle East [23, 32] and Nigeria [16–20]. This ESBL gene has also been incriminated in community outbreaks of multidrug resistant (MDR) *E. coli* infections in some parts of the UK [30] and elsewhere [15].

The literature on the genetic characteristics of ESBLs in Nigeria and, indeed, Africa is sparse. The data emanating so far from Nigeria does not include the prevalence of the CTX-M ESBL in an in-patient setting. With this study we have demonstrated that the prevalence of *Enterobacteriaceae* isolates carrying genes that encode CTX-M-15 ESBL enzymes is at an unacceptable level with potential clinical and financial implications for the hospital.

The clinical implication of this finding is that many patients infected by MDR Gram-negative bacteria stand the risk of treatment failure and cases of fatalities may increase. Thus, treatment of such infections assumes a great challenge to the clinician and the clinical microbiologists as treatment options are limited to very expensive and sometimes toxic drugs. Added to this burden is the fact that the location of the mobile genetic element, *ISEcp1*, a single copy insertion sequence responsible for mobilization of *bla* genes, was found upstream of the *bla*_CTX-M_ genes. This has grave consequences as it might conceivably facilitate the spread of these genes among the *Enterobacteriaceae* within the hospital. Transfer of the *bla*_CTX-M-15_ genes to recipient *E. coli* J53 has been shown to be quite readily achievable. This suggests that resistance genes can easily move from one species to another with the possibility of easy interspecies transfer. One of the limitations of this study was that plasmids were not studied to determine if these genes were the same.

## Conclusion

In conclusion, our study demonstrated an explosive emergence of isolates harboring *bla*_CTX-M-15_ gene mediating CTX-M-type ESBL production in invasive members of family *Enterobacteriaceae*. Immediate implementation of antibiotic stewardship and other preventive strategies are necessary to stem the tide of dangerous spread of MDR *Enterobacteriaceae* in this Lagos hospital.

## Abbreviations

ESBLs: Extended spectrum beta-lactamases
PCR: Polymerase chain reaction
CLSI: Clinical and Laboratory Standard Institute
LASUTH: Lagos State University Teaching Hospital
ICU: Intensive care unit
CCU: Critical care unit
MALDI-TOF: Matrix-assisted laser desorption ionization-time of flight
MS: Mass spectrometry
MIC: Minimum inhibitory concentration
